# Canakinumab treatment shows maintained efficacy in systemic juvenile idiopathic arthritis patients

**DOI:** 10.1186/1546-0096-12-S1-P68

**Published:** 2014-09-17

**Authors:** NM Wulffraat, N Ruperto, HI Brunner, S Oliveira, Y Uziel, K Nistala, R Cimaz, MA Ferrandiz, B Flato, M Gamir, I Kone-Paut, C Gaillez, K Lheritier, K Abrams, A Martini, D Lovell

**Affiliations:** 1 PRINTO-Istituto Gaslini, Genova, Italy; 2 UMC Utrecht, Utrecht, Netherlands; 3 PRCSG, Cincinnati, Ohio, USA; 4 Novartis Pharma AG, Basel, Switzerland; 5 Novartis Pharmaceuticals Corporation, New Jersey, USA

## Introduction

Systemic juvenile idiopathic arthritis (SJIA), an interleukin-1β (IL-1β)-mediated autoinflammatory disease, is characterized by recurrent flares of active disease. Treatment with canakinumab (CAN), a selective, human, anti-IL-1β monoclonal antibody allows for successful steroid dose reduction/discontinuation and reduces risk to experience a flare in patients with SJIA [1]. CAN is approved for SJIA patients (≥ 2 years old) by over 30 countries including USA, EU, Russia and Canada.

## Objectives

To evaluate the maintenance of efficacy with continued CAN treatment in SJIA patients during the blinded randomized treatment withdrawal part of a large phase III trial.

## Methods

Patients 2–19 yrs of age with active SJIA who had responded to open-label CAN treatment 4mg/kg/4wks sc, maintained a minimum adapted ACR Pediatric criteria [aACR] 30 for up to 32 weeks, and were steroid-free or had successfully reduced systemic steroids to a minimum dose, were randomized to either continue CAN or receive placebo until 37 flare events occurred [1]. Patients were considered to have completed the study if they entered clinical remission on medication (CRM), i.e. achieved 24 consecutive weeks of clinical inactive disease (CID) [2]. A survival analysis of the time to worsening in aACR level, after randomization for the CAN and placebo groups was performed. Time to worsening is the time to fail to maintain at least the same level of aACR response seen at randomization. The change in the proportion in each group of those with CID was also evaluated.

## Results

100 pts were randomized to a CAN (n=50) or a placebo (n=50) group, of whom 26 (53%) and 27 (54%), respectively, had CID at the start of the randomization part. In the first 2 months, probability of maintaining aACR response was similar for both treatment groups. Thereafter, the probability of maintaining aACR response was greater in the CAN vs placebo groups. The median time to worsening in aACR level for patients in the placebo group was 141 days (95% CI: 85, 281), and could not be calculated for CAN as <50% of CAN group had a worsening in their aACR level by the end of this phase. The median duration of exposure for the CAN group was 221.5 days (range: 8-617 days). There was a statistically significant relative risk reduction of 51% for the CAN vs placebo group to experience a worsening in aACR level (HR= 0.49; 95% CI: 0.27, 0.90; p=0.0131). CID was achieved by 31(62.0%) vs 17 (34.0%) patients in CAN vs placebo at their last visit (OR= 3.4; 95% CI: 1.5, 8.0; p=0.0020) and CRM was reached by 20 (40%) CAN and 2 (4%) placebo patients by the end of the study.

## Conclusion

A greater proportion of SJIA patients who continued CAN treatment maintained/improved their aACR response, achieved CID and CRM than patients who discontinued CAN by being switched to placebo, demonstrating maintenance of efficacy with continued CAN treatment over time.

## Disclosure of interest

N. Wulffraat Grant / Research Support from: Abbvie, Roche, Consultant for: Novartis, Pfizer, Roche, N. Ruperto Grant / Research Support from: To Gaslini Hospital: Abbott, Astrazeneca, BMS, Centocor Research & Development, Eli Lilly and Company, "Francesco Angelini", Glaxo Smith & Kline, Italfarmaco, Novartis, Pfizer Inc., Roche, Sanofi Aventis, Schwarz Biosciences GmbH, Xoma, Wyeth Pharmaceuticals Inc., Speaker Bureau of: Astrazeneca, Bristol Myers and Squibb, Janssen Biologics B.V., Roche, Wyeth, Pfizer, H. Brunner Consultant for: Novartis, Genentech, Pfizer, UCB, AstraZeneca, Biogen, Boehringer-Ingelheim, Regeneron, Paid Instructor for: Novartis, Speaker Bureau of: Novartis, Genentech, S. Oliveira Grant / Research Support from: Novartis, Roche, Y. Uziel Speaker Bureau of: Fee for few talks at medical meeting- Novartis, Neopharm, Roche, K. Nistala: None declared., R. Cimaz: None declared., M. Ferrandiz Grant / Research Support from: Principal investigator's fee by Novartis, B. Flato Grant / Research Support from: Coinvestigator in the initial study on efficacy by canakinumab treatment in systemic juvenile idiopathic arthritis patients. Expenses for personnel covered by Novartis , M. Gamir: None declared., I. Kone-Paut Grant / Research Support from: SOBI, Chugai, Consultant for: Pfizer, SOBI, Novartis, Chugai, C. Gaillez Employee of: Novartis Pharma AG, K. Lheritier Shareholder of: Novartis, Employee of: Novartis Pharma AG, K. Abrams Shareholder of: Novartis, Employee of: Novartis Pharmaceuticals corporation, A. Martini Grant / Research Support from: Bristol Myers and Squibb, Centocor Research & Development,Glaxo Smith & Kline, Novartis, Pfizer Inc, Roche, Sanofi Aventis, Schwarz Biosciences GmbH, I declare that the Gaslini Hospital which is the public Hospital where I work as full time employee has received contributions to support the PRINTO research activities from the industries above mentioned. OLD: Francesco Angelini S.P.A., Janssen Biotech Inc, Abbott., Consultant for: Bristol Myers and Squibb, Centocor Research & Development,Glaxo Smith & Kline, Novartis, Pfizer Inc, Roche, Sanofi Aventis, Schwarz Biosciences GmbH, I declare that the Gaslini Hospital which is the public Hospital where I work as full time employee has received contributions to support the PRINTO research activities from the industries above mentioned., Speaker Bureau of: Abbott, Bristol Myers Squibb, Astellas, Boehringer, Italfarmaco, MedImmune, Novartis, NovoNordisk, Pfizer, Sanofi, Roche, Servier, D. Lovell Grant / Research Support from: National Institutes of Health- NIAMS , Consultant for: Astra-Zeneca, Centocor, Amgen, Bristol Meyers Squibb, Abbott, Pfizer, Regeneron, Roche, Novartis, UBC, Forest Research Institute, Horizon, Johnson & Johnson, Speaker Bureau of: Novartis, Roche.
